# Efficacy and safety of SGLT2 inhibitors in elderly patients with type 2 diabetes

**DOI:** 10.1080/07853890.2026.2696636

**Published:** 2026-07-13

**Authors:** Chieh-Sen Chuang, Chew-Teng Kor, Shu-Yi Wang, Ying-Lin Hsu

**Affiliations:** a Department of Neurology, Changhua Christian Hospital, Changhua, Taiwan; b Department of Applied Mathematics, National Chung Hsing University, Taichung, Taiwan; c Big Data Center, Changhua Christian Hospital, Taiwan; d Graduate Institute of Clinical Medicine, College of Medicine, National Chung Hsing University, Taichung, Taiwan; e Division of Endocrinology and Metabolism, Department of Internal Medicine, Changhua Christian Hospital, Changhua, Taiwan; f Institute of Statistics, National Chung Hsing University, Taichung, Taiwan; g General Education Center, Chang Jung Christian University, Tainan, Taiwan

**Keywords:** SGLT2 inhibitors, elderly, type 2 diabetes, renal protection, kidney function, eGFR

## Abstract

**Objectives:**

To evaluate the effectiveness and safety of sodium-glucose co-transporter 2 (SGLT2) inhibitors in elderly patients with type 2 diabetes mellitus, with particular focus on renal and cardiovascular outcomes.

**Methods:**

This retrospective cohort study analyzed data from 9,915 diabetic patients aged ≥65 years who received antihyperglycemic therapy at Changhua Christian Hospital, Taiwan, between January 2021 and September 2023. Patients were categorized as SGLT2 inhibitor users (*n* = 3,345) or non-users (*n* = 6,570). After 1:1 propensity score matching, 1,529 patients remained in each group. Primary outcomes included renal function (measured by eGFR decline), coronary artery disease, ischemic stroke, and heart failure. Secondary outcomes included urinary tract infection, genital infection, diabetic ketoacidosis, and hypoglycemia.

**Results:**

SGLT2 inhibitor use was associated with significant renoprotective effects, demonstrated by reduced risk of 30% eGFR decline (HR 0.69, 95% CI 0.59–0.80, *p* < 0.001) and 50% eGFR decline (HR 0.60, 95% CI 0.45–0.80, *p* < 0.001). Subgroup analyses revealed that renoprotective effects were more pronounced in patients with higher baseline eGFR (≥50 mL/min/1.73 m^2^), suggesting greater benefit with early initiation of SGLT2 inhibitors for kidney protection. However, SGLT2 inhibitor use was associated with an increased risk of genital infections (HR 4.29, 95% CI 1.02–18.04, *p* = 0.047) in patients without prior history of such infections.

**Conclusions:**

In elderly patients with type 2 diabetes, SGLT2 inhibitors demonstrate significant renal protective effects, particularly among those with preserved renal function (eGFR ≥50 mL/min/1.73 m^2^). These findings highlight the importance of considering patient characteristics when evaluating potential benefits of SGLT2 inhibitor therapy. The differential risk patterns suggest that clinicians should consider individual patient profiles and medical histories when prescribing these medications. The occurrence of major adverse cardiovascular events (MACE) did not exhibit a statistically significant difference between the cohort administered SGLT2 inhibitors and the cohort not receiving SGLT2 inhibitors (log-rank *p*-value = 0.160). Conversely, the recurrence of MACE was markedly reduced in the cohort receiving SGLT2 inhibitors (log-rank *p*-value < 0.001). These findings should be interpreted in the context of a single-center retrospective design and the inherent limitations of propensity-score-matched observational analyses.

## Introduction

Sodium-glucose co-transporter 2 (SGLT2) inhibitors are a new type of oral antidiabetic drug used to treat type 2 diabetes. These medications work by inhibiting the activity of the SGLT2 protein in the kidneys [1,2]. The SGLT2 protein is responsible for reabsorbing glucose in the kidneys. By blocking this transporter protein, SGLT2 inhibitors prevent glucose from being reabsorbed into the bloodstream, thereby increasing glucose excretion in the urine and lowering blood glucose levels [3,4]. Unlike other types of antidiabetic drugs, the mechanism of action of SGLT2 inhibitors does not rely on insulin secretion or sensitivity. This makes them a valuable treatment option, especially for patients with insufficient insulin secretion or poor response to other medications.

SGLT2 inhibitors have multiple benefits in addition to lowering blood sugar, making them a treatment option for patients with type 2 diabetes [5]. These benefits include weight loss and lower blood pressure [6]. Additionally, large clinical trials have shown that SGLT2 inhibitors may also reduce the risk of major cardiovascular events (such as heart attack and stroke), hospitalization for heart failure, and worsening of chronic kidney disease [4,7]. These benefits, coupled with their unique mechanism of action, make SGLT2 inhibitors a promising treatment option. But they also come with some potential side effects, such as an increased risk of genital infections and diabetic ketoacidosis [2,8]. However, elderly patients are often underrepresented in clinical trials[9]. To better understand the efficacy and safety of SGLT2 inhibitors in the elderly population, additional clinical data are needed [10–12].

This study compares the effectiveness and safety of SGLT2 inhibitor treatment in elderly patients with type 2 diabetes. This study will be arranged with a retrospective trial using the Changhua Christian Hospital Research Database. The safety of SGLT2 inhibitors was evaluated using adverse reaction records. Efficacy assessment was performed for patients with hemoglobin A1c (HbA1c) levels and those treated for more than 12 weeks. Additionally, we will conduct subgroup analyses to explore the effectiveness of SGLT2 inhibitors in patients with varying medical conditions. Through this study, we hope to provide more medical evidence for clinicians when selecting diabetes treatment regimens.

## Method

### Study populations

This retrospective observational cohort study was executed between January 2021 and September 2023, encompassing all patients aged 65 years or older diagnosed with diabetes mellitus who received continuous oral antihyperglycemic therapy at the outpatient facilities of Changhua Christian Hospital (CCH), Taiwan. The study data were sourced from the Clinical Research Database (CCHRD) of CCH, which encompasses a comprehensive array of electronic medical records, including clinical visit documentation, prescriptions, laboratory findings, and hospitalization records [13]. All personally identifiable information was anonymized prior to disclosure. Consequently, the institutional review board waived the necessity for informed consent. We excluded patients with incomplete medical records or those who had been on oral antihyperglycemic therapy for fewer than 90 days. The diagnosis of diabetes mellitus was established based on recognized clinical criteria and validated through a thorough review of medical records.

### Data collection and definition

**Figure 1.**
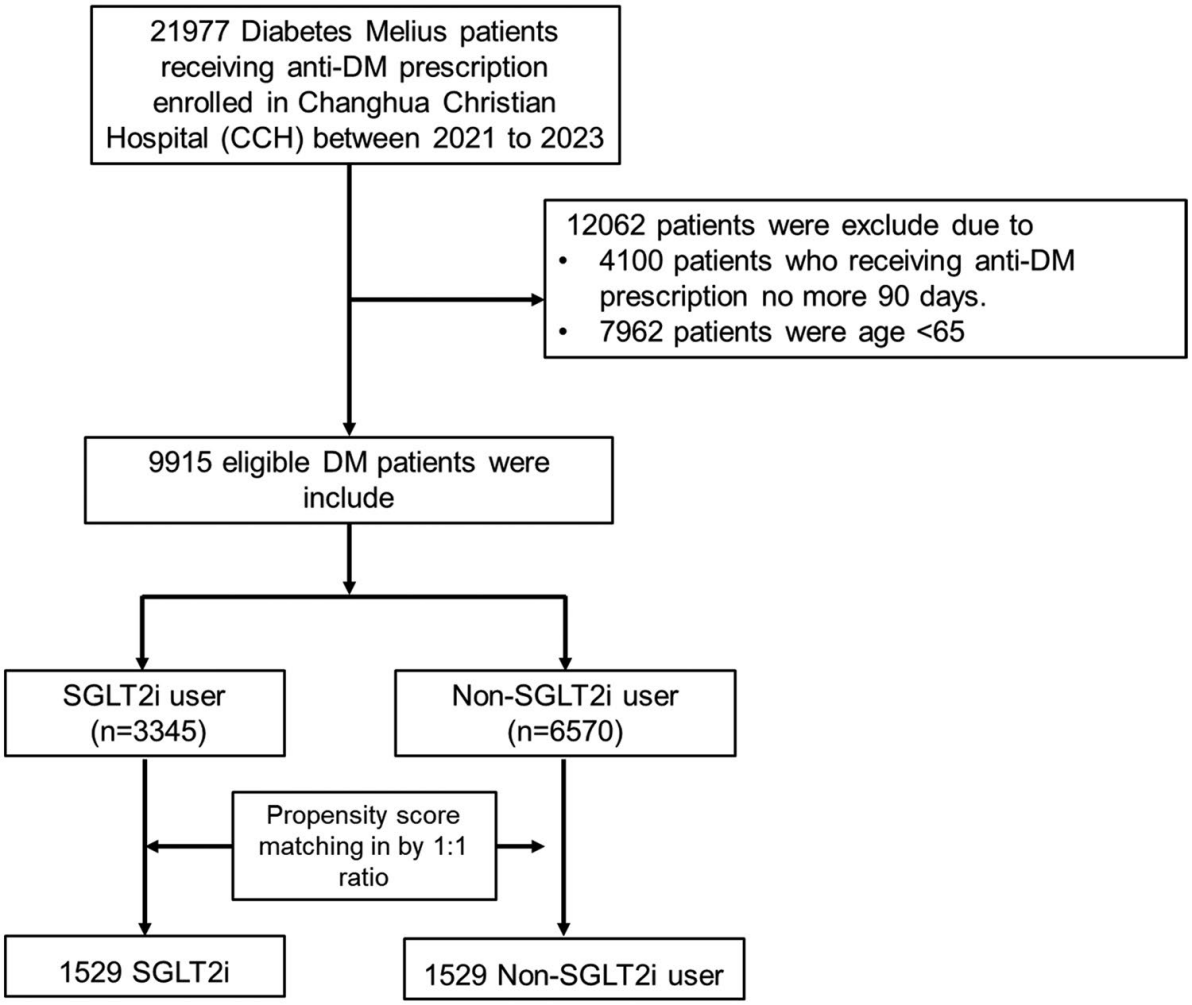
Flow chart.

We analyzed a cohort of 21,977 individuals diagnosed with diabetes mellitus (DM) at Changhua Christian Hospital between January 2021 and September 2023. The data pertaining to these patients were extracted from the electronic medical records maintained by Changhua Christian Hospital. Ultimately, our investigation encompassed 9,915 cases of DM (as depicted in Figure 1). The subsequent data were extracted from medical records: demographic variables (age, gender), duration of diabetes mellitus, comorbid conditions (hypertension, dyslipidemia), prior occurrences of cerebrovascular disease, cardiovascular disease, or heart failure, and the utilization of antihypertensive agents or statins. The laboratory parameters encompassed estimated glomerular filtration rate (eGFR), glycated hemoglobin (HbA1c), total cholesterol, levels of low-density lipoprotein (LDL) cholesterol, high-density lipoprotein (HDL) cholesterol, and the albumin-to-creatinine ratio (ACR). This retrospective study was approved by the Institutional Review Board of Changhua Christian Hospital (CCH IRB No.: 231113). The requirement for written informed consent was waived owing to the retrospective nature of the study and use of de-identified data, in accordance with the CIOMS (Council for International Organizations of Medical Sciences) International Ethical Guidelines and the Declaration of Helsinki.

### Exposure definition

SGLT2 inhibitor exposure was defined using prescription records in the electronic medical database. Patients were classified as SGLT2 inhibitor users if they had documented prescriptions for an SGLT2 inhibitor surrounding the index date and maintained continuous therapy for at least 90 days. The index date was defined as the date of the first eligible SGLT2 inhibitor prescription for users. For non-users, the index date was defined as the first prescription date of any oral antihyperglycemic agent during the study period. To improve exposure ascertainment, patients with less than 90 days of continuous oral antihyperglycemic therapy were excluded from the study. Because medication adherence could not be directly measured from prescription data, exposure was based on prescription records rather than confirmed ingestion.

### Outcome definitions

Clinical outcomes were identified from hospitalization records using International Classification of Diseases (ICD) codes, with confirmation from the principal diagnosis field. Major adverse cardiovascular events (MACE) were defined as a composite of myocardial infarction, stroke, cardiovascular death, and hospitalization for heart failure. Stroke recurrence, coronary artery disease (CAD) recurrence, and heart failure recurrence were defined as subsequent hospitalizations with the same diagnosis after an initial event. Heart failure was defined based on hospitalization with a principal diagnosis of heart failure.

### Study results

The primary outcomes of this investigation encompassed renal function, coronary artery disease (CAD), ischemic stroke, and heart failure. The estimated glomerular filtration rate (eGFR), computed utilizing the CKD-EPI equation, served as the measure for renal function. Secondary outcomes comprised urinary tract infection (UTI), genital infection, diabetic ketoacidosis (DKA), and hypoglycemia. All outcomes were meticulously evaluated from the point of enrollment until the conclusion of the follow-up period. The index date was established as the initial prescription date for SGLT2 inhibitors for users of these agents, and the first prescription date of any oral anti-diabetic medication for non-SGLT2 users. Patient data were censored at the last available follow-up date.

### Statistical analysis

Data are presented as numbers (percentages) for categorical variables and as medians with interquartile ranges (IQRs) for continuous variables. The chi-square test was used to compare categorical data, while the Mann–Whitney U test was applied to compare continuous data between users and non-users. Propensity score matching was employed to mitigate possible confounding factors in the context of this observational investigation. The propensity score was operationalized as the likelihood of being administered SGLT2 inhibitors, contingent upon the observed baseline characteristics. Logistic regression was used to estimate propensity scores, including the following covariates: age, sex, comorbidities (hypertension, hyperlipidemia, Incidence of cardiovascular events, stroke), and baseline laboratory values (eGFR, HbA1c, cholesterol, Albumin to Creatinine Ratio). Patients receiving SGLT2 inhibitors were matched 1:1 to those receiving Oral hypoglycemic agents using a nearest-neighbor algorithm with a caliper width of 0.1 standard deviation of the logit of the propensity score.

Cox’s proportional hazards models were employed to assess the association between the administration of SGLT2 inhibitors and a range of clinical endpoints. Crude hazard ratios (cHR) in conjunction with 95% confidence intervals (95% CI) were calculated utilizing a univariate Cox proportional hazards model. Adjusted Cox proportional hazards models were employed on both the initial and propensity score-matched datasets to augment the reliability of the results. Adjusted hazard ratios (aHR) were derived from multivariate Cox PH models, which considered confounding variables, encompassing all factors specified in Table 1. Propensity score-matched hazard ratios (PSM) were derived utilizing the propensity score-matched dataset, incorporating adjustments for the propensity score. To evaluate covariate balance after matching, standardized mean differences (SMDs) were calculated for all baseline characteristics. An SMD > 0.10 was considered indicative of meaningful imbalance. The distribution of SMDs before and after PSM is presented in Supplementary Table S1. The proportional hazards (PH) assumption was verified for each outcome using a time-dependent covariates approach, in which an interaction term between SGLT2 inhibitor use and follow-up time was incorporated into the Cox model; a statistically significant interaction coefficient was considered evidence of PH violation, and analyses were revised accordingly for affected outcomes. To address residual confounding from covariates remaining imbalanced after PSM (HbA1c and ACR), two additional sensitivity analyses were conducted: (1) a PSM-based Cox model further adjusted for HbA1c and ACR (PSM*), and (2) an inverse probability weighting (IPW) Cox model adjusting for all covariates listed in Table 1 (Supplementary Table S2).

**Table 1.** Demographic and clinical variables.

	Overall patients			Propensity scores matched dataset		
	Non-SGLT2 user	SGLT2 user	*p*-value	Non-SGLT2 user	SGLT2 user	*p*-value
Sample size	6570	3345		1529	1529	
Age, years	75.8 ± 7.6	73.3 ± 6.6	<0.001	72.71 ± 6.37	72.93 ± 6.17	0.338
Age category						
65–74	3304(50.3%)	2158(64.5%)	<0.001	1051(68.7%)	1018(66.6%)	0.017
75–84	2243(34.1%)	946(28.3%)		374(24.5%)	432(28.3%)	
≥85	1023(15.6%)	241(7.2%)		104(6.8%)	79(5.2%)	
Gender						
Female	3667(55.8%)	1574(47.1%)	<0.001	765(50%)	782(51.1%)	0.539
Male	2903(44.2%)	1771(52.9%)		764(50%)	747(48.9%)	
DM duration, years	7.1 ± 4.1	8.2 ± 4.0	<0.001	8.51 ± 3.05	8.4 ± 3.28	0.369
DM duration						
<1	981(14.9%)	307(9.2%)	<0.001	52(3.4%)	54(3.5%)	0.077
2–3	564(8.6%)	266(8%)		87(5.7%)	122(8%)	
4–5	592(9%)	231(6.9%)		108(7.1%)	96(6.3%)	
>5	4433(67.5%)	2541(76%)		1282(83.8%)	1257(82.2%)	
Comorbidity						
Hypertension	5176(78.8%)	2639(78.9%)	0.898	1240(81.1%)	1242(81.2%)	0.926
Hyperlipidemia	4715(71.8%)	2731(81.6%)	<0.001	1341(87.7%)	1341(87.7%)	1.000
Mace	3703(56.4%)	1921(57.4%)	0.061	785(51.3%)	822(53.8%)	0.180
Stroke	2250(34.2%)	837(25%)	<0.001	343(22.4%)	377(24.7%)	0.147
CAD	2100(32%)	1369(40.9%)	<0.001	575(37.6%)	584(38.2%)	0.737
HF	686(10.4%)	450(13.5%)	<0.001	144(9.4%)	141(9.2%)	0.852
Medication use						
Insulin	1054(16%)	822(24.6%)	<0.001	374(24.5%)	390(25.5%)	0.504
Anti-HTN drug	896(13.6%)	474(14.2%)	0.467	218(14.3%)	206(13.5%)	0.530
Statin	5023(76.5%)	2927(87.5%)	<0.001	1375(89.9%)	1386(90.6%)	0.502
Lab data						
eGFR	68.05(47.84,86.22)	69.01(50.76,86.26)	0.004	71.82(52.1,89.08)	71.11(53.82,86.57)	0.675
HbA1c	6.7(6.2,7.4)	7.3(6.7,8.1)	<0.001	7.0(6.5,7.9)	7.2(6.6,7.9)	<0.001
LDL cholesterol	76(61,94)	75(61,91)	0.031	75(60,91)	0.75(62,90)	0.628
Cholesterol	141(122,162)	139(122,160)	0.246	140(122,160)	140(122,159)	0.773
HDL cholesterol	42.5(36,51)	44(37,52)	<0.001	44(37,53)	44(37,52)	0.703
ACR	27.7(11.9,126.9)	33.1(14.4,128.1)	<0.001	23.7(10.7,106.4)	31.1(14.2,115.1)	<0.001
Propensity score	0.31 ± 0.15	0.42 ± 0.16	<0.001	0.4 ± 0.16	0.4 ± 0.16	0.977

Abbreviations: SGLT2i: sodium-glucose cotransporter 2 inhibitor; DM: diabetes mellitus; T2DM: type 2 diabetes mellitus; eGFR: estimated glomerular filtration rate; HbA1c: glycated hemoglobin; LDL: low-density lipoprotein; HDL: high-density lipoprotein; ACR: albumin-to-creatinine ratio; CAD: coronary artery disease; CVD: cardiovascular disease; HF: heart failure; HTN: hypertension.

Kaplan-Meier methodologies were employed to assess and graphically represent the cumulative incidence functions across various treatment cohorts. All statistical evaluations were performed utilizing SAS version 9.4 and R software (version 4.1.0). A *p*-value <0.05 was considered statistically significant in this study.

## Result

### Patient characteristics before and after propensity score matching

A total of 21,977 diabetes mellitus patients receiving anti-diabetic medication prescriptions were enrolled at Changhua Christian Hospital (CCH) between 2021 and 2023. Of these, 12,062 patients were excluded from the study: 4,100 patients due to receiving anti-diabetic medication prescriptions for less than 90 days, and 7,962 patients who were younger than 65 years old. This resulted in 9,915 eligible diabetic patients for inclusion in the study.

Among the eligible patients, 3,345 were identified as SGLT2 inhibitor users, while 6,570 were non-SGLT2 inhibitor users. To minimize potential confounding factors and selection bias, propensity score matching was applied with a 1:1 ratio. This matching process resulted in two equal groups of 1,529 patients each: one group of SGLT2 inhibitor users and another group of non-SGLT2 inhibitor users (Figure 1).

Before Propensity Score Matching, significant differences were observed between the two groups in several baseline characteristics: SGLT2 inhibitors (SGLT2i) users were younger, SGLT2i users had a higher proportion of males, SGLT2 inhibitors users had longer diabetes duration. In comorbidities, SGLT2i users had higher rates of hyperlipidemia, CAD, and heart failure. After Propensity Score Matching, 1,529 patients were included in each group. Most baseline characteristics were well-balanced between the groups, with no statistically significant differences except HbA1c and ACR. Propensity score matching successfully balanced most baseline characteristics between SGLT2i users and non-users, allowing for a more robust comparison of outcomes between the two groups.

Standardized mean differences (SMDs) for all covariates before and after PSM are displayed in Supplementary Table S1. Although most covariates achieved adequate balance post-matching (SMD < 0.10), HbA1c and ACR remained relatively imbalanced (SMD > 0.10). Sensitivity analyses using PSM* (additionally adjusted for HbA1c and ACR) and IPW Cox models yielded results consistent with the primary analysis, supporting the robustness of our findings (Supplementary Table S2). Regarding competing risks, death was considered as a competing event in the propensity score-matched cohort. However, the mortality proportion was low (121 deaths among 3,058 matched patients; 3.96%), which was below the 5% threshold, suggesting that the impact of competing risk was limited and unlikely to materially affect the main findings.

Based on the Kaplan–Meier curves presented in Figure 2, the following results can be reported for the comparison between SGLT2 inhibitors and non-SGLT2 inhibitors in diabetic patients aged 65 and older.

Figure 2.Kaplan–Meier curve for incidence and recurrence of MACE outcome: Cumulative incidence of cardiovascular outcomes in elderly patients with type 2 diabetes treated with SGLT2 inhibitors versus non-users. Panels A and B show major adverse cardiovascular events (MACE); C and D, stroke; E and F, coronary artery disease (CAD); and G and H, heart failure (HF). Log-rank tests were used for group comparisons. This figure displays cumulative incidence of cardiovascular events in elderly patients (≥65 years) with type 2 diabetes. A–B: Major Adverse Cardiovascular Events (MACE). While incident MACE showed no significant difference between treatment groups (*p* = 0.160), SGLT2 inhibitor users demonstrated significantly lower MACE recurrence (*p* < 0.001). C–D: Stroke outcomes. SGLT2 inhibitor users exhibited significantly reduced risk for both stroke incidence (*p* = 0.019) and recurrence (*p* = 0.005). E–F: Coronary Artery Disease (CAD). The non-SGLT2 inhibitor group showed significantly lower CAD incidence (*p* = 0.001), while CAD recurrence rates were comparable between groups (*p* = 0.674). G–H: Heart Failure. Neither heart failure incidence (*p* = 0.823) nor recurrence (*p* = 0.357) differed significantly between treatment groups.

**Figure:**
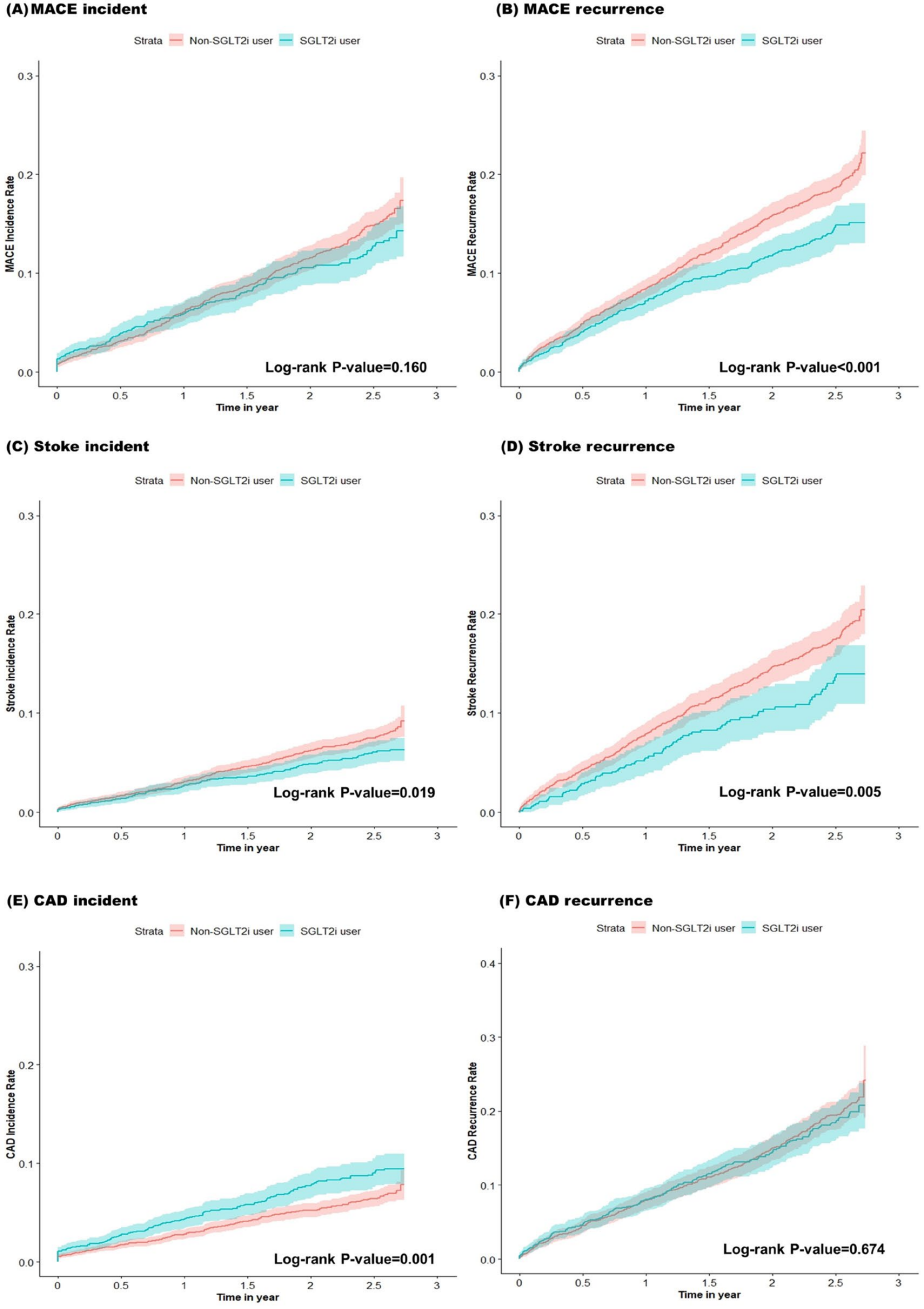

**Figure:**
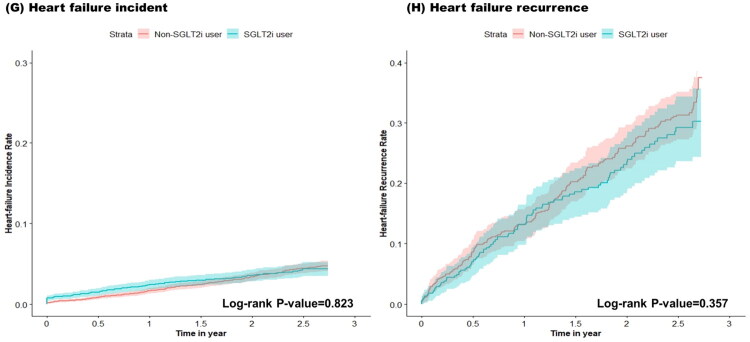

### Major cardiovascular events

MACE Outcomes: The incidence of MACE did not differ significantly between the SGLT2 inhibitor group and the non-SGLT2 inhibitor group (log-rank *p*-value = 0.160). However, MACE recurrence was significantly lower in the SGLT2 inhibitor group (log-rank *p*-value < 0.001) (Figure 2A, 2B).

Stroke: Both stroke incidence and recurrence were significantly lower in the SGLT2 inhibitor group compared to the non-SGLT2 inhibitor group (incidence: log-rank *p*-value = 0.019; recurrence: log-rank *p*-value = 0.005). (Figure 2C, 2D)

CAD (Coronary Artery Disease): The incidence of CAD was significantly lower in the non-SGLT2 inhibitor group (log-rank *p*-value = 0.001). However, there was no significant difference in CAD recurrence between the two groups (log-rank *p*-value = 0.674) (Figure 2E, 2F).

Heart Failure: There was no significant difference between the SGLT2 inhibitor and non-SGLT2 inhibitor groups in either the incidence (log-rank *p*-value = 0.823) or recurrence (log-rank *p*-value = 0.357) of heart failure (Figure 2G, 2H).

**Figure 3.**
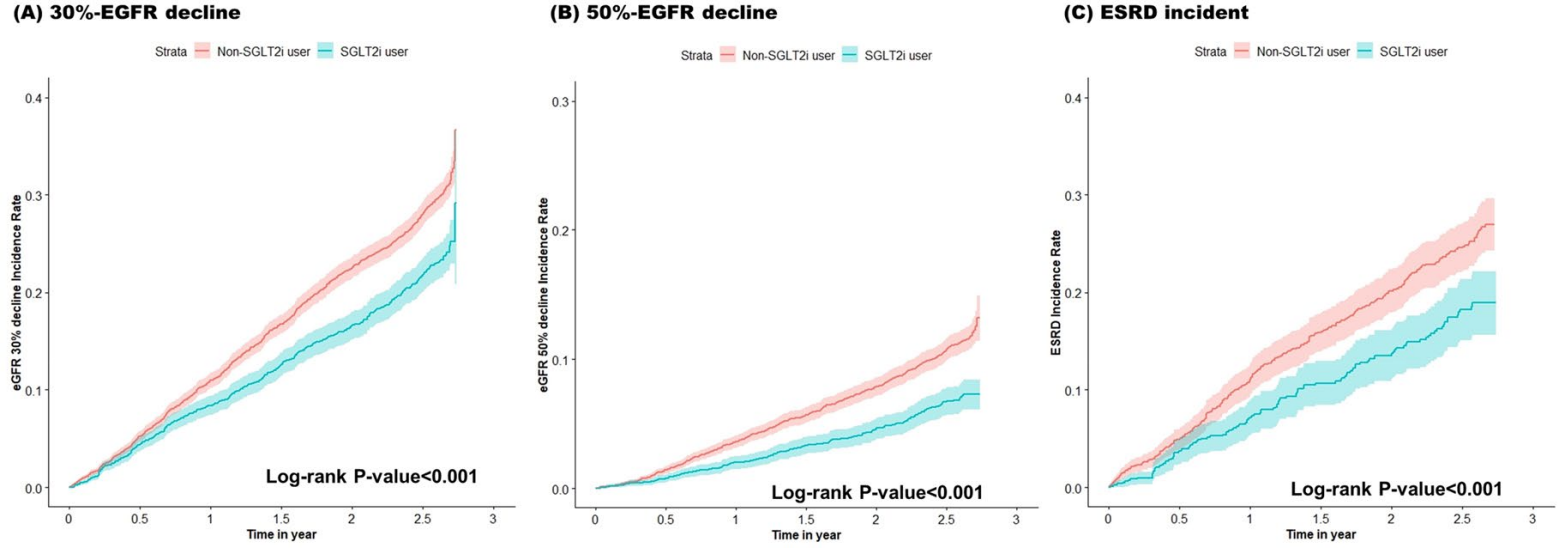
Kaplan–Meier curve for renal outcome: Cumulative incidence of renal outcomes in elderly patients with type 2 diabetes treated with SGLT2 inhibitors versus non-users. Panels A, B, and C show 30% eGFR decline, 50% eGFR decline, and end-stage renal disease (ESRD), respectively. Log-rank tests were used for group comparisons. This figure depicts the renal outcomes in elderly patients (≥65 years) with type 2 diabetes. (A) Incidence of 30% eGFR decline: The SGLT2 inhibitor group (blue line) showed a consistently lower cumulative incidence of 30% eGFR decline compared to the non-SGLT2 inhibitor group (pink line) throughout the 3-year follow-up (log-rank *p* < 0.001). (B) Incidence of 50% eGFR decline: SGLT2 inhibitor users also demonstrated a reduced cumulative incidence of 50% eGFR decline (log-rank *p* < 0.001). (C) ESRD incidence: The cumulative incidence of end-stage renal disease (ESRD) was significantly lower in the SGLT2 inhibitor group (blue line) compared to the non-SGLT2 inhibitor group (red line) over the 3-year period (log-rank *p* < 0.001), indicating a substantial reduction in progression to ESRD among SGLT2 inhibitor users. eGFR: estimated glomerular filtration rate.

The Kaplan–Meier curves for renal outcomes in patients aged 65 years and older with diabetes mellitus are presented in Figure 3. The graphs illustrate the cumulative incidence of eGFR decline over a period of approximately 3 years, comparing SGLT2 inhibitor users to non-SGLT2 inhibitor users.

Figure 3A depicts the incidence of 30% eGFR decline. The SGLT2 inhibitor group (blue line) demonstrated a consistently lower cumulative incidence of 30% eGFR decline compared to the non-SGLT2 inhibitor group (pink line) throughout the follow-up period. The difference between the two groups was statistically significant (log-rank *p*-value <0.001). Figure 3B shows the incidence of 50% eGFR decline, representing a more severe deterioration in renal function. SGLT2 inhibitor users exhibited a lower cumulative incidence of 50% eGFR decline compared to non-SGLT2 inhibitor users. This difference was also statistically significant (log-rank *p*-value <0.001). Figure 3C illustrates the cumulative incidence of End-Stage Renal Disease (ESRD) over a 3-year follow-up period, comparing elderly patients (≥65 years) with type 2 diabetes treated with SGLT2 inhibitors (blue line) versus non-users (red line). The graph demonstrates a significantly lower risk of progression to ESRD in the SGLT2 inhibitor group compared to the non-SGLT2 inhibitor group (log-rank *p*-value < 0.001). It represented a clinically reduction in the severe renal outcome among elderly patients with type 2 diabetes receiving SGLT2 inhibitor therapy.

**Figure 4.**
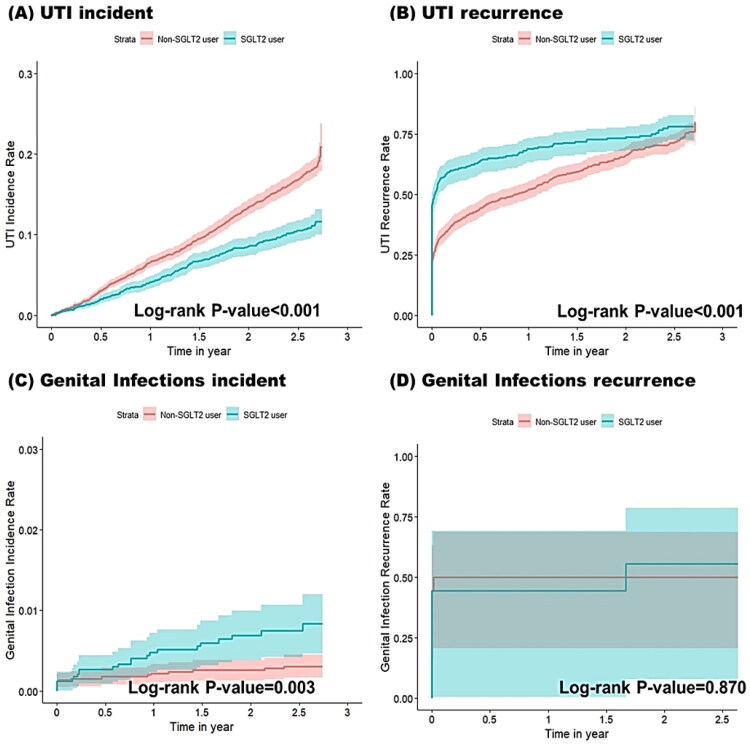
Kaplan–Meier curve for incidence and recurrence of DM-related complications: Cumulative incidence of urinary tract and genital infections in elderly patients with type 2 diabetes treated with SGLT2 inhibitors versus non-users. Panels A and B show urinary tract infection (UTI) incidence and recurrence, respectively; C and D show genital infection incidence and recurrence, respectively. Log-rank tests were used for group comparisons. This figure describes the urinary tract infections (UTI) and genital infections in elderly patients with type 2 diabetes. (A) New-Onset UTI Incidence: SGLT2 inhibitor users demonstrated a consistently lower incidence of new UTI compared to non-users throughout the 3-year follow-up period (log-rank *p* < 0.001). (B) UTI Recurrence: SGLT2 inhibitor users showed a higher UTI recurrence rate compared to non-users throughout the 3-year follow-up period (log-rank *p* < 0.001). (C) Genital Infections Incident: SGLT2 inhibitor users exhibited a significantly higher incidence of new genital infections (log-rank *p* = 0.003). (D) Genital Infection Recurrence: No statistically significant difference was observed in genital infection recurrence between the two groups (log-rank *p* = 0.870), with similar recurrence rates and substantial overlap in confidence intervals.

For urinary tract infections (UTIs), both incident cases and recurrences showed statistically significant differences between the two groups (log-rank *p* < 0.001 for both). The incidence of new-onset UTIs was lower in SGLT2 inhibitor users compared to non-users throughout the 3-year follow-up period (Figure 4A). However, for UTI recurrence, SGLT2 inhibitor users showed a higher rate compared to non-users (Figure 4B).

Regarding genital infections, there was a significant difference in the incidence of new cases between SGLT2 inhibitor users and non-users (log-rank *p* = 0.003, Figure 4C). However, for the recurrence of genital infections, no statistically significant difference was observed between the two groups (log-rank *p* = 0.870, Figure 4D). The recurrence rates for both groups appeared to be similar, with substantial overlap in the confidence intervals throughout the study period.

In the propensity score-matched analysis of SGLT2 inhibitor use in older adults with diabetes (≥65 years), several significant outcomes were observed (Table 2):

**Table 2.** An assessment of the impact of SGLT2 on different clinical outcomes using univariate and multivariate Cox regression models.

Outcome	cHR (95% CI)	*p*-value	aHR (95% CI)	*p*-value	PSM (95% CI)	*p*-value
DM-related complication						
UTI event						
UTI incident	0.60(0.52,0.69)	<0.001	0.69(0.57,0.84)	<0.001	0.77(0.61,0.97)	0.026
UTI recurrent	1.29(1.11,1.49)	0.001	1.41(1.12,1.76)	0.003	1.15(0.88,1.50)	0.320
Genital infection						
Genital infection incident	3.97(1.77,8.91)	0.001	2.92(1.07,7.94)	0.036	4.29(1.02,18.04)	0.047
Genital infection recurrent	1.13(0.38,3.37)	0.829	1.03(0.33,3.25)	0.957	0.96(0.21,4.27)	0.951
DKA event						
DKA incident	0.55(0.30,1.02)	0.058	0.72(0.28,1.86)	0.496	0.49(0.15,1.57)	0.229
DKA recurrent	1.02(0.77,1.36)	0.892	1.20(0.81,1.78)	0.359	1.39(0.86,2.24)	0.177
Glycemic control						
Hypoglycemic	0.93(0.70,1.25)	0.644	1.25(0.85,1.84)	0.262	1.12(0.67,1.87)	0.664
Renal outcome						
30%-eGFR decline	0.74(0.68,0.82)	<0.001	0.79(0.71,0.89)	<0.001	0.69(0.59,0.80)	<0.001
50%-eGFR decline	0.59(0.49,0.70)	<0.001	0.75(0.60,0.92)	0.007	0.60(0.45,0.80)	<0.001
ESRD	0.68(0.56,0.82)	<0.001	0.73(0.55,0.96)	0.026	0.81(0.56,1.17)	0.262
MACE outcome						
Overall MACE						
MACE incident	0.86(0.71,1.04)	0.119	1.10(0.86,1.40)	0.432	1.03(0.74,1.44)	0.861
MACE recurrent	0.74(0.63,0.87)	<0.001	0.94(0.76,1.17)	0.587	0.92(0.68,1.25)	0.609
Stroke						
Stroke incident	0.76(0.61,0.94)	0.013	0.98(0.75,1.30)	0.914	1.02(0.70,1.50)	0.909
Stroke recurrent	0.70(0.55,0.89)	0.003	0.73(0.53,1.00)	0.053	0.76(0.49,1.17)	0.216
CAD						
CAD incident	1.41(1.15,1.73)	0.001	1.46(1.11,1.93)	0.007	1.22(0.82,1.81)	0.329
CAD recurrent	0.96(0.81,1.14)	0.616	1.04(0.83,1.29)	0.746	1.16(0.86,1.55)	0.331
Heart failure						
Heart failure incident	1.03(0.81,1.30)	0.834	1.48(1.06,2.06)	0.022	1.3(0.81,2.09)	0.274
Heart failure recurrent	0.88(0.68,1.12)	0.298	1.32(0.91,1.92)	0.139	1.1(0.68,1.77)	0.714

Abbreviations: SGLT2i: sodium-glucose cotransporter 2 inhibitor; cHR: crude hazard ratio; aHR: adjusted hazard ratio; CI: confidence interval; PSM: propensity score-matched; UTI: urinary tract infection; DKA: diabetic ketoacidosis; eGFR: estimated glomerular filtration rate; MACE: major adverse cardiovascular events; CAD: coronary artery disease; HF: heart failure; HbA1c: glycated hemoglobin; LDL: low-density lipoprotein; HDL: high-density lipoprotein.

Diabetes-related complications: SGLT2 inhibitor use was associated with a 23% lower risk of incident urinary tract infections (UTIs) (HR 0.77, 95% CI 0.61–0.97, *p* = 0.026). However, there was a significantly increased risk of incident genital infections (HR 4.29, 95% CI 1.02–18.04, *p* = 0.047).

Renal outcomes: SGLT2 inhibitor use was associated with a significant reduction in the risk of 30% eGFR decline (HR 0.69, 95% CI 0.59–0.80, *p* < 0.001) and 50% eGFR decline (HR 0.60, 95% CI 0.45–0.80, *p* < 0.001). Although the risk of unadjusted ESRD was significant reduction (*p* < 0.001). After propensity-score matching and multivariable adjustment, the association between SGLT2 inhibitor use and incident ESRD was no longer statistically significant (Table 2).

The subgroup analysis in Table 3 demonstrated the impact of SGLT2 inhibitors on various clinical outcomes:

**Table 3.** Subgroup analysis.

Outcome subgroup	Age < 75	Age ≥ 75	*P*_interact_	eGFR < 50	eGFR ≥ 50	*P*_interact_	LDL < 100	LDL ≥ 100	*P*_interact_
DM-related complication									
UTI event									
UTI incident	0.74(0.55,1.00)	0.79(0.55,1.13)	0.793	0.89(0.67,1.17)	0.61(0.4,0.91)	0.129	0.83(0.65,1.06)	0.54(0.29,0.98)	0.195
UTI recurrent	1.24(0.88,1.74)	0.95(0.61,1.48)	0.352	1.30(0.96,1.77)	0.70(0.39,1.26)	0.067	1.15(0.85,1.55)	1.16(0.63,2.14)	0.970
Genital infection									
Genital infection incident	3.53(0.8,15.52)	4.71(0.12,192.25)	0.888	3.18(0.72,13.97)	7.32(0.18,305.7)	0.684	5.60(0.9,34.89)	1.93(0.19,20.03)	0.483
Genital infection recurrent	0.66(0.12,3.69)	UE		UE	0.95(0.15,6.00)		1.51(0.28,8.04)	0.16(0.01,15.27)	0.366
DKA event									
DKA incident	1.01(0.14,7.19)	0.33(0.07,1.52)	0.380	0.60(0.06,6.27)	0.58(0.15,2.20)	0.972	0.42(0.12,1.55)	1.08(0.07,17.26)	0.548
DKA recurrent	1.68(0.86,3.29)	1.11(0.55,2.23)	0.399	1.39(0.77,2.51)	1.58(0.67,3.70)	0.812	1.29(0.78,2.15)	1.83(0.35,9.45)	0.691
Glycemic control									
Hypoglycemic	1.10(0.56,2.18)	1.10(0.51,2.38)	0.995	1.23(0.62,2.41)	1.08(0.49,2.38)	0.814	1.18(0.68,2.06)	0.88(0.24,3.28)	0.691
Renal outcome									
30%-eGFR decline	0.63(0.51,0.77)	0.74(0.59,0.94)	0.290	0.82(0.67,0.99)	0.51(0.4,0.66)	0.004	0.69(0.59,0.82)	0.68(0.46,0.99)	0.903
50%-eGFR decline	0.63(0.44,0.91)	0.54(0.35,0.84)	0.609	0.85(0.57,1.28)	0.45(0.3,0.67)	0.027	0.6(0.44,0.82)	0.62(0.31,1.21)	0.950
ESRD	0.99(0.63,1.55)	0.52(0.26,1.04)	0.125	0.87(0.58,1.31)	0.57(0.23,1.38)	0.394	0.79(0.52,1.2)	0.89(0.38,2.1)	0.815
MACE outcome									
Overall MACE									
MACE incident	0.82(0.53,1.26)	1.42(0.82,2.48)	0.124	1.18(0.79,1.74)	0.73(0.37,1.44)	0.237	1.24(0.85,1.81)	0.49(0.21,1.11)	0.042
MACE recurrent	1.05(0.69,1.61)	0.80(0.52,1.23)	0.363	1.24(0.84,1.84)	0.60(0.36,0.99)	0.025	0.99(0.71,1.39)	0.72(0.37,1.43)	0.414
Stroke									
Stroke incident	0.93(0.55,1.57)	1.07(0.61,1.91)	0.720	1.02(0.66,1.58)	1.02(0.45,2.30)	0.987	1.13(0.74,1.73)	0.66(0.26,1.68)	0.309
Stroke recurrent	0.83(0.47,1.45)	0.69(0.35,1.37)	0.689	0.88(0.53,1.46)	0.50(0.21,1.23)	0.284	0.92(0.57,1.49)	0.32(0.11,0.96)	0.085
CAD									
CAD incident	0.98(0.61,1.56)	2.23(0.99,5.03)	0.086	1.22(0.76,1.96)	1.33(0.64,2.77)	0.847	1.52(0.97,2.38)	0.51(0.19,1.32)	0.042
CAD recurrent	0.97(0.66,1.43)	1.46(0.93,2.29)	0.183	1.54(1.06,2.22)	0.68(0.41,1.12)	0.011	1.13(0.82,1.57)	1.31(0.67,2.55)	0.702
Heart failure									
Heart failure incident	1.16(0.54,2.46)	1.31(0.71,2.4)	0.804	2.73(1.33,5.61)	0.60(0.29,1.21)	0.003	1.68(0.99,2.85)	0.39(0.11,1.41)	0.039
Heart failure recurrent	1.36(0.66,2.80)	0.91(0.47,1.76)	0.423	1.70(0.81,3.54)	0.79(0.40,1.57)	0.139	1.01(0.58,1.75)	1.54(0.57,4.14)	0.461

Abbreviations: SGLT2i: sodium-glucose cotransporter 2 inhibitor; eGFR: estimated glomerular filtration rate; MACE: major adverse cardiovascular events; CAD: coronary artery disease; LDL: low-density lipoprotein; UTI: urinary tract infection; DKA: diabetic ketoacidosis; UE: unestimated due to the small number of cases. Subgroup analyses were exploratory and not adjusted for multiple comparisons.

Renal Outcomes: In the subgroup analysis stratified by baseline eGFR, SGLT2 inhibitors showed a more pronounced protective effect against 30% eGFR decline in patients with eGFR ≥50 mL/min/1.73m^2^ (aHR 0.51, 95% CI 0.40–0.66) compared to those with eGFR <50 mL/min/1.73 m^2^ (aHR 0.82, 95% CI 0.67–0.99), with a significant interaction (*p*-interaction = 0.004). Similarly, for 50% eGFR decline, SGLT2 inhibitors demonstrated greater efficacy in the eGFR ≥50 group (aHR 0.45, 95% CI 0.30–0.67) versus the eGFR <50 group (aHR 0.85, 95% CI 0.57–1.28), with *p*-interaction = 0.027.

Cardiovascular Outcomes: SGLT2 inhibitors were associated with a reduced risk of recurrent MACE in patients with eGFR ≥50 mL/min/1.73 m^2^ (aHR 0.60, 95% CI 0.36–0.99) but not in those with eGFR <50 mL/min/1.73 m^2^ (aHR 1.24, 95% CI 0.84–1.84), with a significant interaction (*p*-interaction = 0.025).

In patients with lower renal function (eGFR <50), SGLT2 inhibitors were associated with an increased risk of CAD recurrence (aHR 1.54, 95% CI 1.06–2.22). Conversely, in patients with higher renal function (eGFR ≥50), SGLT2 inhibitors showed a trend towards protection against CAD recurrence, although not statistically significant (aHR 0.68, 95% CI 0.41–1.12). The interaction between eGFR status and SGLT2 inhibitor effect was statistically significant (p-interaction = 0.011), indicating that renal function may modify the impact of SGLT2 inhibitors on CAD recurrence risk.

SGLT2 inhibitors were associated with an increased risk of incident heart failure in patients with eGFR <50 mL/min/1.73m^2^ (aHR 2.73, 95% CI 1.33–5.61) but not in those with eGFR ≥50 mL/min/1.73m^2^ (aHR 0.60, 95% CI 0.29–1.21), with a significant interaction (*p*-interaction = 0.003).

In the LDL cholesterol subgroup analysis, SGLT2 inhibitors showed a lower MACE incident in patients with LDL ≥100 mg/dL (aHR 0.49, 95% CI 0.21–1.11), compared with LDL <100 mg/dL (aHR 1.24, 95% CI 0.85–1.81), with *p*-interaction = 0.042.

These findings suggest that the effects of SGLT2 inhibitors on various cardiovascular and renal outcomes may differ based on baseline eGFR and LDL cholesterol levels in older adults with type 2 diabetes.

## Discussion

Our study provides insights into the efficacy and safety of SGLT2 inhibitors in elderly diabetic patients aged 65 and above. The results demonstrate both benefits and potential risks associated with SGLT2 inhibitor use in this population. SGLT2 inhibitors showed remarkable renoprotective effects, with significant reductions in 30% eGFR decline (HR 0.69, 95% CI 0.59–0.80) and 50% eGFR decline (HR 0.60, 95% CI 0.45–0.80). Interestingly, subgroup analyses revealed that the renoprotective effects were more pronounced in patients with higher baseline eGFR (≥50 mL/min/1.73 m^2^), suggesting that early initiation of SGLT2 inhibitors may offer greater benefit in preserving renal function. Notably, we observed a higher incidence of genital infections among SGLT2 inhibitor users (HR 4.29, 95% CI 1.02–18.04, *p* = 0.047), consistent with the known side effect profile of this drug class. Furthermore, our findings indicate potential cardiovascular benefits, particularly in specific subgroups. SGLT2 inhibitors were associated with a reduced risk of MACE recurrence in patients with eGFR ≥50 mL/min/1.73 m^2^ (HR 0.60, 95% CI 0.36–0.99). Conversely, we observed an increased risk of recurrent CAD (HR 1.54, 95% CI 1.06–2.22) and new onset of heart failure (HR 2.73, 95% CI 1.33–5.61) in patients with eGFR < 50 mL/min/1.73 m^2^, highlighting the complex interplay between SGLT2 inhibitors and cardiovascular outcomes in elderly patients.

Beyond the glucose-lowering effects, SGLT2 inhibitors have demonstrated a range of other benefits, including modest weight loss and potential cardio-renal protection [14,15]. A meta-analysis including 26,106 participants with diabetic kidney disease from 8 large-scale trials found that a 34% reduction in the risk of kidney composite outcomes (HR 0.66, 95% CI: 0.58–0.75), such as sustained decline in estimated glomerular filtration rate (eGFR), progression to end-stage kidney disease, or renal death [16]. The renal benefits of SGLT2 inhibitors are derived from a combination of synergistic hemodynamic and metabolic pathways that collectively preserve renal function. By enhancing sodium delivery to the macula densa, SGLT2 inhibitors trigger afferent arteriolar constriction, which mitigates glomerular hyperfiltration [17,18]. These agents attenuate the activation of the intrarenal renin-angiotensin-aldosterone system (RAAS), providing an additional mechanism to reduce hyperfiltration [18]. Reducing glucose reabsorption in the proximal tubule diminishes local metabolic demand and oxygen consumption, thereby improving renal cortical oxygenation [19]. The J-CKD-DB study showed that SGLT2 inhibitor initiation slowed eGFR decline versus other glucose‑lowering agents in patients with type 2 diabetes and CKD, with consistent benefit across age strata, suggesting that renoprotective efficacy is not modified by age [20]. In our cohort of patients aged 65 years and older with diabetes mellitus, SGLT2 inhibitor use over approximately three years was associated with significantly lower incidences of 30% and 50% eGFR decline compared with other antidiabetic drugs, indicating protection against progression of renal dysfunction in this elderly population. Moreover, subgroup analyses demonstrated that these renoprotective effects were more pronounced in patients with baseline eGFR at least 50 mL/min/1.73 m^2^, implying that earlier initiation of SGLT2 inhibitors in patients with preserved renal function may confer greater long‑term kidney benefit.

The recently published 2026 AHA/ACC/ADA/ASN Guideline for the Prevention, Detection, Evaluation, and Management of Cardiovascular-Kidney-Metabolic (CKM) Syndrome provides a novel staging framework that underscores the interconnected nature of type 2 diabetes (T2D), chronic kidney disease (CKD), and cardiovascular disease (CVD) [21]. CKM syndrome staging classifies individuals along a continuum from stage 0 (no risk factors) through stage 4 (established CVD with metabolic or kidney comorbidities), with stages 2 and 3 – defined by the presence of T2D and/or CKD with or without subclinical CVD – carrying substantially elevated risks for atherosclerotic cardiovascular disease, heart failure, atrial fibrillation, and all-cause mortality. The majority of elderly patients with T2D in our cohort, many of whom exhibited impaired renal function, would likely fall within CKM stages 2 to 3, the very population in whom the guideline assigns a Class I recommendation for SGLT2 inhibitor use – both to reduce loss of kidney function and to lower the risks of HF hospitalization and cardiovascular mortality. The guideline further specifies that among adults with CKM stage 2–3, T2D, and a 10-year predicted CVD risk ≥7.5%, the treatment plan should include an SGLT2 inhibitor or GLP-1–based therapy with demonstrated cardiovascular benefit.

SGLT2 inhibitors have different effects on the risk of stroke in elderly diabetic patients in various studies. P. Tsai et al. searched four databases for randomized clinical trials (RCTs) until November 2022 and enrolled 79,504 patients with type 2 diabetes from 43 studies[22]. They found no difference in the risk of ischemic stroke among patients who took an SGLT2 inhibitor compared to those who took a placebo or oral hypoglycemic drugs. The risk ratio (RR) was 0.95, with a 95% confidence interval of 0.79–1.14. Subgroup analysis revealed that none of the SGLT2 inhibitor treatments (canagliflozin, dapagliflozin, empagliflozin, and ertugliflozin) significantly altered outcomes when analyzed separately. A recent study explored the age-dependent impact of SGLT2 inhibitors on stroke risk among elderly patients with diabetes mellitus (DM) and atrial fibrillation (AF) [23]. Using a longitudinal cohort of 9,669 patients from a single medical center database spanning 2010 to 2020, participants were stratified into three distinct age groups (<75, 75–89, and ≥90 years) to evaluate differential effects of SGLT2i therapy on stroke outcomes. In patients younger than 75 years, SGLT2i use was associated with a significant reduction in stroke risk (HR 0.63, 95% CI 0.44–0.88, *p* < 0.05). In the 75 to 89-year-old group, SGLT2i therapy appeared to exert a neutral effect on stroke risk (HR 0.95, 95% CI 0.60–1.50), suggesting that the protective benefits observed in younger patients may diminish with advancing age. Notably, in patients aged 90 and above, SGLT2 inhibitors were associated with an increased risk of stroke (HR 5.04, 95% CI 1.20–21.1, *p* < 0.05). According to our study, during the three-year follow-up period, the incidence of both new-onset and recurrent stroke was lower in patients receiving SGLT2 inhibitors compared to those not treated with these agents (Figure 2C and 2D). However, after adjustment for potential confounding factors, this difference did not reach statistical significance (Table 2). Furthermore, subgroup analyses stratified by age (<75 years vs. ≥75 years) and renal function (eGFR <50 vs. ≥50 mL/min/1.73 m^2^) demonstrated no statistically significant differences in stroke incidence between SGLT2 inhibitor users and non-users within these subgroups (Table 3). These findings suggest that while there may be a trend toward reduced stroke risk with SGLT2i treatment, the benefit is not statistically confirmed across different age and renal function categories in our cohort.

Numerous cardiovascular outcome studies have investigated the impact of SGLT2 inhibitors on cardiovascular outcomes in individuals diagnosed with type 2 diabetes mellitus. The EMPA-REG OUTCOME study assessed the influence of empagliflozin on cardiovascular outcomes in patients with T2DM who also have established cardiovascular disease [24]. The trial demonstrated that empagliflozin significantly reduced the risk of MACE (major adverse cardiovascular events), as well as cardiovascular death and hospitalization for heart failure. The DECLARE-TIMI 58 trial conducted an evaluation of the impact of dapagliflozin on cardiovascular outcomes within a more extensive cohort of individuals diagnosed with type 2 diabetes mellitus (T2DM), encompassing participants with pre-existing cardiovascular conditions as well as those exhibiting numerous cardiovascular risk factors [25]. While dapagliflozin did not significantly reduce the risk of MACE in the overall population, it did significantly reduce the risk of hospitalization for heart failure. The CANVAS Program evaluated the effects of canagliflozin on cardiovascular and renal outcomes in patients with T2DM and established cardiovascular disease or multiple cardiovascular risk factors [8,26]. The CANVAS Program demonstrated that canagliflozin markedly diminished the incidence of major adverse cardiovascular events (MACE), in addition to reducing the likelihood of hospitalization due to heart failure. In our investigation involving geriatric patients diagnosed with diabetes, the occurrence of recurrent major adverse cardiovascular events (MACE) throughout a three-year follow-up interval was lower among individuals administered SGLT2 inhibitors in comparison to the control cohort. (Figure 2B). However, after adjustment for potential confounders, this difference did not reach statistical significance (Table 2). Notably, subgroup analysis revealed that in patients with preserved renal function (eGFR ≥ 50 mL/min/1.73 m^2^), the incidence of recurrent MACE was significantly lower in the SGLT2 inhibitor group compared to controls (Table 3). These findings suggest that renal function may modify the cardiovascular benefits of SGLT2 inhibitors in elderly diabetic patients.

The cardiovascular findings of the present study warrant careful comparison with randomized controlled trials, particularly DECLARE–TIMI 58. While DECLARE–TIMI 58 demonstrated that dapagliflozin did not significantly reduce MACE incidence (HR 0.93; 95% CI 0.84–1.03; *p* = 0.17) but did reduce hospitalization for heart failure in a broad adult population [25], our study similarly found no significant reduction in incident MACE (*p* = 0.861), yet demonstrated a significant reduction in recurrent MACE specifically among patients with preserved renal function (eGFR ≥50 mL/min/1.73 m^2^; aHR 0.60, 95% CI 0.36–0.99). Several important methodological and population-level factors may account for these divergent patterns. First, DECLARE–TIMI 58 enrolled a heterogeneous population with a mean age of approximately 64 years, of whom 59% had only multiple cardiovascular risk factors rather than established atherosclerotic cardiovascular disease, yielding a comparatively lower baseline cardiovascular event rate [25]; by contrast, our cohort comprised exclusively elderly patients (≥65 years), who are characterized by higher cardiovascular comorbidity burden, greater polypharmacy, and an inherently elevated risk of recurrent vascular events. Second, the mechanisms underlying SGLT2 inhibitor cardioprotection – including natriuresis-mediated reduction in preload, attenuation of sympathetic activation, and improvement in myocardial energetics – may manifest differently with advancing age, when arterial stiffness, impaired baroreceptor sensitivity, and reduced renal reserve alter the hemodynamic response to glucosuria-driven volume shifts [15]. Third, DECLARE–TIMI 58 excluded the majority of patients with eGFR below 60 mL/min/1.73 m^2^ (only 7% at randomization) [25], whereas our study enrolled patients across a wide range of renal function, and our subgroup analyses demonstrated that cardiovascular benefit was largely confined to those with eGFR ≥50, consistent with the premise that sufficient proximal tubular SGLT2 activity is required to generate meaningful hemodynamic and metabolic cardioprotection. Fourth, the divergent findings regarding incident versus recurrent MACE in our study may reflect the enriched secondary-prevention profile of elderly real-world patients, among whom the absolute risk reduction from recurrent events is more readily detectable over a shorter observation period. Finally, differences in ethnic composition – our cohort being exclusively East Asian – as well as variations in background cardiovascular pharmacotherapy and glycemic control targets between a clinical trial setting and routine outpatient care may further contribute to the observed heterogeneity of cardiovascular outcomes across studies.

The mechanism behind SGLT2 inhibitors involves increased glycosuria, which could theoretically raise urinary tract infections (UTIs) risk. A meta-analysis of randomized controlled trials has examined the association between SGLT2 inhibitors and the risk of UTIs. No significant difference in UTIs was observed between SGLT2 inhibitors and control groups (Risk Ratio [RR] 1.05, 95% Confidence Interval [CI] 0.98–1.12) [27]. However, a retrospective cohort study suggests that SGLT2Is may lower the risk of UTIs compared to dipeptidyl peptidase 4 inhibitors, showing a hazard ratio of 0.90, 95% Confidence Interval: 0.83–0.98 [28]. Additionally, a pan-India study found that while the incidence of UTIs is higher in the early months of SGLT2I use, it decreases with prolonged use, indicating adaptation over time [29]. Our study found that during the three-year follow-up, the incidence of new-onset urinary tract infections progressively decreased in patients using SGLT2 inhibitors. However, among patients with a prior UTI history, those using SGLT2 inhibitors demonstrated a higher rate of recurrent UTIs (Figure 2). After multivariate adjustment, the overall risk of UTI incidence was significantly reduced (HR = 0.77, 95% CI 0.61–0.97). Subgroup analyses demonstrated that patients with preserved renal function (eGFR ≥ 50 mL/min/1.73 m^2^) and LDL cholesterol ≥ 100 mg/dL exhibited particularly lower UTI rates compared to controls. The finding of a lower incidence of urinary tract infection among SGLT2 inhibitor users should be interpreted cautiously, as it is not entirely consistent with prior literature. Several explanations may account for this observation. First, detection bias may have influenced the results, because patients receiving SGLT2 inhibitors may have had closer clinical monitoring, which could affect the ascertainment of infection outcomes. Second, patient selection may have played a role, as clinicians may have been more likely to prescribe SGLT2 inhibitors to patients perceived to be at lower risk for infection or with more favorable baseline clinical profiles. In addition, the use of retrospective administrative data may have led to misclassification of mild or uncoded UTI events, and the relatively low number of events may have limited statistical precision. Therefore, although our data suggest a lower risk of incident UTI in this cohort, this association should be considered exploratory and hypothesis-generating rather than definitive evidence of a protective effect.

SGLT2 inhibitors are correlated with an elevated risk of genital infections owing to their pharmacological action of facilitating glucose excretion *via* urine, thereby establishing a conducive environment for the proliferation of fungi. A systematic review encompassing pivotal clinical trials, such as CANVAS and EMPA-REG, underscored a greater frequency of genitourinary infections (GUI) among patients administered SGLT2 inhibitors in comparison to those receiving placebo, with 2.24% of the treated cohort reporting GUI as opposed to 0.40% within the placebo cohort [30]. A retrospective cohort study assessed the risk of genital infections linked to SGLT2 inhibitors versus other antidiabetic medications, such as DPP-4 inhibitors and GLP1 agonists, using data from two US commercial claims databases (2013–2017). SGLT2 inhibitors are linked to approximately a three-fold higher risk of genital infections compared to other anti-diabetic medications such as DPP-4 inhibitors (Adjusted Hazard Ratios (HR): Females: 2.81, Males: 2.68) [31]. A study analyzed the risk of genital infections in 221,004 type 2 diabetes patients starting SGLT2i and 55,471 controls starting DPP4i using a UK primary care database. Patients with a history of genital infections are more likely to experience recurrent infections when using SGLT2 inhibitors. Women with prior infections have a particularly high incidence rate (23.7%) compared to those without prior infections (10.8%) [32]. Providing hygiene advice can significantly reduce the incidence of genital infections in patients on SGLT2 inhibitors. In one study, only 4.8% of patients who followed hygiene practices developed infections, compared to 40.8% of those who did not [33]. Our findings found a complex relationship between SGLT2 inhibitor use and the risk of genital infections over a three-year follow-up period. Among elderly DM patients without a prior history of genital infections, those using SGLT2 inhibitors experienced a significantly higher incidence of new-onset genital infections compared to the control group (Figure 4, Table 2). In contrast, for patients with a history of genital infections, the rate of recurrent infections did not differ significantly between the SGLT2 inhibitor group and the control group. This finding suggests that while SGLT2 inhibitors may increase the risk of first-time genital infections, their impact on recurrence rates in high-risk individuals may be less pronounced in elderly DM patients. The incidence of genital infections may be impacted by whether clinical physicians engage in proactive health education in elderly people with DM.

Beyond the core outcomes examined in the present study, several emerging lines of evidence offer complementary perspectives on the broader management of type 2 diabetes mellitus, particularly with respect to oxidative stress, rare adverse events, comparative drug efficacy, and integrative therapeutic strategies. From a pathophysiological standpoint, the regulation of selenoproteins has been proposed as a novel avenue for mitigating metabolic and oxidative disturbances in diabetes. Selenoproteins, which are essential enzymes for maintaining redox balance, play a pivotal role in modulating insulin biosynthesis, endoplasmic reticulum stress, and glucose metabolism; notably, both selenium deficiency and excess exert adverse effects on insulin signaling, underscoring the importance of optimal rather than maximal selenium intake, while targeted suppression of Selenoprotein P has shown promise in ameliorating insulin resistance in preclinical models[34]. These antioxidant-based mechanistic insights may complement the cardio-renal protective pathways attributed to SGLT2 inhibitors in elderly diabetic patients, though their direct clinical applicability in this population warrants further investigation. Regarding the safety profile of SGLT2 inhibitors, acute pancreatitis represents a rare but clinically significant adverse event that merits attention as prescriptions for this drug class continue to rise. A systematic review and case analysis by Li et al. identified 21 cases of SGLT2 inhibitor-induced acute pancreatitis – predominantly associated with empagliflozin – with a median onset of 21 days after drug initiation; reassuringly, all patients recovered fully following drug discontinuation and conservative management, suggesting a generally favorable prognosis when the condition is recognized promptly [35]. This finding reinforces the importance of vigilant pharmacovigilance, particularly in elderly patients who may present atypically or harbor competing etiologies for pancreatitis. Furthermore, comparative efficacy data between drug classes in ethnically specific populations are of growing relevance. A preliminary head-to-head study in Chinese patients with type 2 diabetes demonstrated that dulaglutide (1.5 mg weekly) achieved superior glycemic control, as reflected by greater HbA1c reduction, compared with dapagliflozin (10 mg daily) over 26 weeks, albeit at the cost of a higher incidence of gastrointestinal adverse events; no serious events necessitating treatment discontinuation were reported in either group[36]. These ethnic-specific findings highlight the heterogeneity in treatment response that may influence clinical decision-making when selecting between GLP-1 receptor agonists and SGLT2 inhibitors in Chinese and other Asian populations, a consideration that deserves attention when contextualizing the present study’s findings. Finally, mounting evidence supports the adjunctive role of herbal medicine in modulating gut-mediated metabolic pathways in diabetes. Astragalus (Astragali Radix) and its bioactive constituents – including polysaccharides, saponins, and flavonoids – have been shown to restore intestinal barrier integrity, regulate gut microbiota composition, attenuate systemic inflammation, and alleviate insulin resistance through modulation of short-chain fatty acid production, bile acid metabolism, and lipopolysaccharide-mediated immune activation [37]. While these preclinical and translational findings are promising, their application as adjuncts to SGLT2 inhibitor therapy in elderly diabetic cohorts remains to be validated in well-designed clinical trials. Taken together, these complementary perspectives – from antioxidant-based interventions and rare adverse event profiling to ethnic-specific efficacy comparisons and gut-targeted herbal adjuncts – collectively enrich the understanding of integrative risk mitigation strategies in the management of type 2 diabetes, and may help inform future research directions that extend beyond the primary scope of the present investigation.

SGLT2 inhibitors, dipeptidyl peptidase-4 (DPP4) inhibitors, and glucagon-like peptide-1 (GLP-1) receptor agonists represent three important classes of glucose-lowering medications for type 2 diabetes management, yet they demonstrate fundamentally different safety profiles and cardiovascular, renal protective effects. In a network meta-analysis, SGLT-2 inhibitors significantly reduced cardiovascular (RR = 0.88) and total (RR = 0.87) death, as compared with DPP-4 inhibitors. The comparison between GLP-1RA and SGLT-2 inhibitors showed no difference in their risks of MACE, nonfatal MI, nonfatal stroke, CV and total death[38]. DPP4 inhibitors have demonstrated neutral cardiovascular effects; they neither significantly reduce nor increase the risk of major adverse events, establishing them as a safe but less cardioprotective option compared to the newer agents[39]. In head-to-head comparisons within heart failure populations, SGLT2 inhibitors were associated with a lower risk of hospitalization for heart failure compared to DPP4 inhibitors (hazard ratio 0.65) and GLP-1 receptor agonists (0.89), with benefits consistent across both reduced and preserved ejection fraction phenotypes [40]. Renal protection provides another dimension where these agents differ substantially. In a direct comparative study, SGLT2 inhibitor initiation was associated with a lower 5-year risk of chronic kidney disease progression (6.7% vs. 8.2% for GLP-1 receptor agonists) and a lower 5-year mean cumulative count of acute kidney injury events (25.2 vs. 28.7 per 100 individuals) [41].

The cardiovascular findings should be interpreted cautiously in the context of prior randomized trials. In contrast to the consistent cardiovascular benefit reported in several large trials, our results showed a mixed pattern for incident and recurrent MACE, as well as an increased risk of heart failure in certain subgroups. These discrepancies may reflect differences in population characteristics, including the advanced age of our cohort, higher baseline cardiovascular risk, and the real-world clinical setting of this retrospective study. In addition, residual confounding and selection bias may have persisted despite propensity score matching, and the relatively small number of events together with the limited follow-up duration may have further affected the stability of the estimates. Accordingly, these cardiovascular findings should be regarded as exploratory and interpreted with caution.

The subgroup analyses should be interpreted as exploratory rather than confirmatory. Although we observed possible effect modification by baseline eGFR and LDL cholesterol in some outcomes, these findings should be viewed with caution because multiple subgroup comparisons were performed without formal adjustment for multiplicity. In addition, subgroup analyses are inherently more vulnerable to reduced statistical power, sparse events, and chance findings, particularly in a retrospective observational study. Therefore, the observed heterogeneity in treatment effects may generate hypotheses for future research, but it should not be interpreted as definitive evidence of differential efficacy or harm across subgroups. Further prospective studies with prespecified subgroup hypotheses and appropriate correction for multiple comparisons are needed to validate these observations.

Our study has several limitations that should be acknowledged. First, as a retrospective observational study using data from a single medical center, the findings may not be generalizable to other populations or healthcare settings. Second, despite propensity score matching to balance baseline characteristics between groups, residual confounding from unmeasured variables cannot be ruled out. Third, the follow-up period of approximately three years may be insufficient to capture the long-term effects of SGLT2 inhibitors, particularly on cardiovascular outcomes that may take longer to manifest. Furthermore, inherent time-related biases of our retrospective, single-center cohort design and predefined three-year observation window should be taken into account. Because exposure to SGLT2 inhibitors was defined according to prescription records within this fixed period, we cannot exclude immortal time bias or time-lag bias, whereby patients had to survive and remain under observation long enough to initiate SGLT2 inhibitor therapy, potentially leading to an underestimation of risks in the exposed group compared with non-users. Secular changes in prescribing behavior, background cardiometabolic management, and diagnostic intensity over the study period may also have differentially influenced the likelihood of detecting outcomes between groups. Although we applied propensity score matching and multivariable adjustment to balance baseline characteristics, residual confounding by time-varying factors such as changes in renal function, comorbidity burden, and concomitant medications is likely to persist. Future prospective studies with longer follow-up periods and more diverse populations are needed to confirm our findings and address these limitations.

## Conclusion

In this retrospective cohort study of older adults with type 2 diabetes, SGLT2 inhibitor use was associated with favorable renal outcomes and an increased risk of genital infections, while the cardiovascular findings were mixed and should be interpreted cautiously. The observed associations may reflect the influence of residual confounding, selection bias, and the characteristics of this real-world elderly population. Further prospective studies with longer follow-up and more rigorous control of confounding are needed to better clarify the cardiovascular effects of SGLT2 inhibitors in older patients.

## Supplementary Material

Supplemental material_2026_06_22a.docx

## Data Availability

The data that support the findings of this study are not publicly available because this information could compromise the privacy of research participants. The data will be shared on reasonable request to the corresponding author (Ying-Lin Hsu, Shu-Yi Wang).
