# Supplementary material for: Efficacy and safety of SGLT2 inhibitors in elderly patients with type 2 diabetes
Source: Ann Med. 2026 Jul 13;58(1):2696636. doi: 10.1080/07853890.2026.2696636 (PMC13366639; doi:10.1080/07853890.2026.2696636)
Supplement: Supplemental material_2026_06_22a.docx [file IANN_A_2696636_SM1897.docx]

Supplementary Material

Table S1: Standardized mean differences (SMDs) for all baseline covariates before and after propensity score matching are shown; an SMD < 0.10 was considered to indicate adequate balance.

|  | Propensity scores matched dataset | | | |
| --- | --- | --- | --- | --- |
|  | non-SGLT2 user | SGLT2 user | P-value | SMD |
| Sample size | 1529 | 1529 |  |  |
| Age, year | 72.71±6.37 | 72.93±6.17 | 0.338 | 0.035 |
| Age category |  |  |  |  |
| 65-74 | 1051(68.7%) | 1018(66.6%) | 0.017 | 0.046 |
| 75-84 | 374(24.5%) | 432(28.3%) |  |  |
| ≥ 85 | 104(6.8%) | 79(5.2%) |  |  |
| Gender |  |  |  |  |
| Female | 765(50%) | 782(51.1%) | 0.539 | 0.022 |
| Male | 764(50%) | 747(48.9%) |  |  |
| DM duration, year | 8.51±3.05 | 8.4±3.28 | 0.369 | 0.036 |
| DM duration |  |  |  |  |
| <1 | 52(3.4%) | 54(3.5%) | 0.077 | 0.007 |
| 2-3 | 87(5.7%) | 122(8%) |  |  |
| 4-5 | 108(7.1%) | 96(6.3%) |  |  |
| >5 | 1282(83.8%) | 1257(82.2%) |  |  |
| Comorbidity |  |  |  |  |
| Hypertension | 1240(81.1%) | 1242(81.2%) | 0.926 | 0.003 |
| Hyperlipidemia | 1341(87.7%) | 1341(87.7%) | 1.000 | 0.000 |
| Mace | 785(51.3%) | 822(53.8%) | 0.180 | 0.048 |
| Stroke | 343(22.4%) | 377(24.7%) | 0.147 | 0.052 |
| CAD | 575(37.6%) | 584(38.2%) | 0.737 | 0.012 |
| HF | 144(9.4%) | 141(9.2%) | 0.852 | 0.007 |
| Medication use |  |  |  |  |
| Insulin | 374(24.5%) | 390(25.5%) | 0.504 | 0.023 |
| Anti-HTN drug | 218(14.3%) | 206(13.5%) | 0.530 | 0.023 |
| Statin | 1375(89.9%) | 1386(90.6%) | 0.502 | 0.024 |
| Lab data |  |  |  |  |
| eGFR | 71.82(52.1,89.08) | 71.11(53.82,86.57) | 0.675 | 0.006 |
| HbA1c | 7.0(6.5,7.9) | 7.2(6.6,7.9) | <0.001 | 0.054 |
| LDL cholesterol | 75(60,91) | 0.75(62,90) | 0.628 | 0.003 |
| Cholesterol | 140(122,160) | 140(122,159) | 0.773 | 0.006 |
| HDL cholesterol | 44(37,53) | 44(37,52) | 0.703 | 0.027 |
| ACR | 23.7(10.7,106.4) | 31.1(14.2,115.1) | <0.001 | 0.007 |
| Propensity score | 0.4±0.16 | 0.4±0.16 | 0.977 | 0.006 |

Table S2: sensitivity analyses: Sensitivity analyses included the propensity score-matched Cox model additionally adjusted for HbA1c and ACR, as well as the inverse probability weighting (IPW) Cox model adjusted for all covariates listed in Table 1.

| Outcome | PSM* (95% CI) | p-value | IPW (95% CI) | p-value |
| --- | --- | --- | --- | --- |
| DM-related complication |  |  |  |  |
| UTI event |  |  |  |  |
| UTI incident | 0.76(0.6,0.95) | 0.0178 | 0.69(0.62,0.78) | <.0001 |
| UTI recurrent | 1.15(0.87,1.5) | 0.3242 | 1.29(1.1,1.5) | 0.0014 |
| Genital infection |  |  |  |  |
| Genital infection incident | 4.34(1.03,18.22) | 0.045 | 2.23(1.11,4.45) | 0.0237 |
| Genital infection recurrent | 0.94(0.15,5.94) | 0.9459 | 1.33(0.51,3.48) | 0.5572 |
| DKA event |  |  |  |  |
| DKA incident | 0.51(0.16,1.63) | 0.2542 | 0.66(0.34,1.27) | 0.2106 |
| DKA recurrent | 1.45(0.89,2.37) | 0.14 | 1.26(0.96,1.65) | 0.0935 |
| Glycemic control |  |  |  |  |
| Hypoglycemic | 1.12(0.67,1.86) | 0.6747 | 1.36(1.06,1.74) | 0.014 |
| Renal outcome |  |  |  |  |
| 30%-eGFR decline | 0.64(0.55,0.75) | <.0001 | 0.77(0.71,0.83) | <.0001 |
| 50%-eGFR decline | 0.54(0.41,0.72) | <.0001 | 0.76(0.65,0.87) | 0.0001 |
| ESRD | 0.78(0.54,1.14) | 0.1972 | 0.78(0.64,0.96) | 0.0186 |
| MACE outcome |  |  |  |  |
| Overall MACE |  |  |  |  |
| MACE incident |  |  |  |  |
| SGLT2 | 1.8(0.92,3.53) | 0.0869 | 1.77(1.29,2.43) | 0.0005 |
| SGLT2*Time | 0.64(0.41,0.99) | 0.0443 | 0.72(0.58,0.9) | 0.003 |
| MACE recurrent | 0.91(0.68,1.24) | 0.5618 | 0.85(0.73,1) | 0.0525 |
| Stroke |  |  |  |  |
| Stroke incident | 1.01(0.69,1.49) | 0.9452 | 0.95(0.78,1.16) | 0.6374 |
| Stroke recurrent | 0.75(0.49,1.16) | 0.1949 | 0.64(0.51,0.8) | <.0001 |
| CAD |  |  |  |  |
| CAD incident | 1.21(0.81,1.8) | 0.3607 | 1.41(1.15,1.73) | 0.0011 |
| CAD recurrent | 1.16(0.87,1.56) | 0.3193 | 1.01(0.86,1.19) | 0.8959 |
| Heart failure |  |  |  |  |
| Heart failure incident | 1.19(0.74,1.92) | 0.4744 | 1.4(1.1,1.77) | 0.006 |
| Heart failure recurrent | 1.16(0.71,1.88) | 0.5557 | 1.39(1.05,1.83) | 0.0211 |
